# Ageing, Cellular Senescence and Neurodegenerative Disease

**DOI:** 10.3390/ijms19102937

**Published:** 2018-09-27

**Authors:** Marios Kritsilis, Sophia V. Rizou, Paraskevi N. Koutsoudaki, Konstantinos Evangelou, Vassilis G. Gorgoulis, Dimitrios Papadopoulos

**Affiliations:** Laboratory of Histology & Embryology, Medical School, National and Kapodistrian University of Athens, 75 Mikras Asias Street, Goudi, 115-27 Athens, Greece; marios.kritsilis@gmail.com (M.K.); sofiriz92@gmail.com (S.V.R.); pkoutsoudaki@med.uoa.gr (P.N.K.); cnevagel@med.uoa.gr (K.E.)

**Keywords:** neurodegeneration, cellular senescence, ageing, Alzheimer’s disease, multiple sclerosis, Parkinson’s disease, lipofuscin, SenTraGor^TM^ (GL13), senolytics

## Abstract

Ageing is a major risk factor for developing many neurodegenerative diseases. Cellular senescence is a homeostatic biological process that has a key role in driving ageing. There is evidence that senescent cells accumulate in the nervous system with ageing and neurodegenerative disease and may predispose a person to the appearance of a neurodegenerative condition or may aggravate its course. Research into senescence has long been hindered by its variable and cell-type specific features and the lack of a universal marker to unequivocally detect senescent cells. Recent advances in senescence markers and genetically modified animal models have boosted our knowledge on the role of cellular senescence in ageing and age-related disease. The aim now is to fully elucidate its role in neurodegeneration in order to efficiently and safely exploit cellular senescence as a therapeutic target. Here, we review evidence of cellular senescence in neurons and glial cells and we discuss its putative role in Alzheimer’s disease, Parkinson’s disease and multiple sclerosis and we provide, for the first time, evidence of senescence in neurons and glia in multiple sclerosis, using the novel GL13 lipofuscin stain as a marker of cellular senescence.

## 1. Ageing and Neurodegeneration

Ageing is a universal process characterized by the accumulation of biological changes that lead to the organism’s functional decline over time. Human ageing is accompanied by a gradual build-up of cognitive and physical impairment and an increased risk of developing numerous diseases including cancer, diabetes, cardiovascular, musculoskeletal and neurodegenerative conditions. Age-related disability and morbidity adversely affect the quality of life; they are ultimately associated with an increased risk of death and bear dire consequences for the individual, the family and society.

Ageing is the most important risk factor for the development of neurodegenerative disease and typically, most neurodegenerative disorders manifest in the elderly [1]. The annual incidence of Alzheimer’s disease (AD) has been shown to increase exponentially with advancing age [2,3]. Notably, Down syndrome, a progeroid condition, has been associated with AD, and mouse models of premature ageing have been reported to overproduce Aβ and show impaired learning and memory [4,5,6,7]. Incidence of Parkinson’s disease (PD), the second most common age-related neurodegenerative condition also increases with age [8,9]. The great majority of AD and PD cases are sporadic and typically manifest at a much older age than hereditary ones. Despite the differences in pathology among the two conditions, they are both typical neurodegenerative diseases characterized by chronic progressive loss of neurons and their synaptic connections manifesting with gradual functional decline [4]. But age is a recognized risk factor even for inflammatory demyelinating conditions such as multiple sclerosis (MS), which also has a strong neurodegenerative component [10]. Age is the strongest predictor for the transition from the relapsing phase of MS, which is primarily inflammatory to the secondary progressive phase of the disease, which is thought to be mainly neurodegenerative [10,11]. 

Although research on the biology of mammalian ageing has recently attracted much attention, our understanding of its underlying mechanisms remains poor. It has been hypothesized that failure of repair mechanisms leads to accumulation of cellular and molecular damage that drives ageing [12]. Accumulating damage is thought to occur inherently in a random manner, which explains the great diversity in ageing phenotypes, even in monozygotic twins [13]. The interplay among the genetic background, environmental factors and the stochastic nature of age-related accumulation of irreparable damage to the DNA of the organism may also determine the likelihood of developing a particular age-related disease. Genomic instability, telomere attrition, loss of proteostasis, dysregulated nutrient sensing, mitochondrial dysfunction, stem cell exhaustion, altered cellular communication and excessive cellular senescence have all been recognized as hallmarks of ageing [14]. Cellular senescence is a process triggered by irreparable DNA damage that underlies normal ageing. Senescent cells become more abundant with ageing and a growing body of evidence suggests that their accumulation may contribute to pathogenesis of age-related diseases. Here, we review the data that support a role for cellular senescence in neurodegeneration, with special focus on AD, PD and MS. 

## 2. Cellular Senescence

Cellular senescence is a homeostatic response aiming to prevent the propagation of damaged cells and neoplastic transformation [15]. Apart from its beneficial role as an anti-tumour response, physiological roles for cellular senescence have also been identified during development [15,16], in adult megakaryocytes, syncytiotrophoblasts, wound healing and placental natural killer lymphocytes [17,18,19]. However, several lines of evidence indicate that cellular senescence also contributes to the loss of function associated with ageing and age-related disease [20]. According to the original observations by Hayflick and Moorhead (1961), when cultures of normal human fibroblasts were passaged serially they underwent stable cell cycle arrest that was accompanied by stereotypical phenotypic changes [21]. This form of cellular senescence, termed replicative senescence constitutes a particular type of cellular senescence and is associated with telomere shortening with successive cell cycles. Nevertheless, besides telomere shortening, there are many more triggers of cellular senescence including aberrant oncogene activation (oncogene-induced senescence-OIS) [22], stress-induced (stress-induced premature senescence-SIPS) due to oxidative stress [23], ionizing radiation [24], DNA-damaging chemotherapy [25], hyperoxia [26], impaired autophagy [27] or other stressors and mitochondrial dysfunction [28]. Most of these triggers lead to telomeric or non-telomeric DNA damage or altered chromatin structure and typically activate the DNA damage response (DDR) [29,30], although cellular senescence in vitro may also occur without detectable DDR [31,32]. When the cell’s repair mechanisms become overwhelmed, the activated DDR elicits cellular senescence via phosphorylation of p53 [33].

Unlike apoptosis, senescent cells remain viable and metabolically active [30]. Although senescent cells can be recognized by T helper cells and cleared by macrophages and natural killer lymphocytes [27,34,35], their number has been shown to increase with normal ageing in tissues of humans, primates and rodents [36,37]. Models of accelerated cellular senescence show premature ageing and increased incidence of age-related pathologies, suggesting that accumulating senescent cells contributes to ageing-related functional compromise and predisposes to age-related disease [38]. The features of senescent cells that constitute the senescent phenotype may be responsible for their putative detrimental effects in ageing and ageing- associated neurodegenerative disease. 

## 3. The Senescence Phenotype

Senescent cells exhibit is a multitude of cellular and molecular changes that are neither specific nor pathognomonic of the senescent state. Evidence suggests that the cellular and molecular features of senescence depend on both the triggering stimulus and the affected cell type [23]. Although the senescence phenotype of nervous system cells has not been extensively studied, the key features of senescence are described below: 

Typically, senescent cells exhibit permanent cell cycle arrest, which is thought to be regulated by p16^INK4A^ and p53-p21-RB (retinoblastoma). Increased expression of p53 upregulates expression of CDKi(cyclin-dependent kinase inhibitor) p21, which initially arrests the cell cycle. p16^INK4A^ mediates permanent cells cycle arrest by inhibiting CDK4 and CDK6, which leads to RB hypophosphorylation and blocks entry to S phase [39,40]. 

Another key feature of cellular senescence is the senescence-associated secretory phenotype (SASP), which is dependent on p38MAPK (p38 mitogen-activated protein kinases), NF-κB (nuclear factor kappa-light-chain-enhancer of activated B cells), cGAS(cyclic GMP-AMP synthase)/STING(stimulator of IFN genes), NOTCH and mTORmammalian target of rapamycin) signalling [38,39,40,41,42]. SASP consists of chemokines, cytokines, growth factors and metaloproteinases [43]. These factors are primarily proinflammatory and act in a paracrine and autocrine manner [44,45], although immunosuppressive mediators have also been identified as part of SASP [46,47]. Recent data indicate that SASP may also involve small extracellular vesicles in a p53-dependent manner [48,49,50].

Cellular senescence is also characterized by resistance to apoptotic death, which appears to be largely controlled by the p53 stress response pathway. Both p53 levels and p53 post-translational modifications [51] seem to have a role in determining the senescent cell fate while conferring resistance to apoptosis. Accumulation of intermediate levels of p53 has been reported to favor the expression of anti-apoptotic bcl-2 family proteins [52]. Nonetheless, p21 has also been shown to be capable to directly inhibit caspase 3 and apoptosis [53]. 

Cellular senescence is associated with changes in cellular metabolism. These include upregulation of lysosomal senescence-associated β-galactosidase, a shift from oxidative phosphorylation to glycolysis [54,55] and accumulation of lipofuscin in the cytoplasm [56,57]. Lipofuscin accumulation has been reported as a key feature of cellular senescence that can be used to positively identify senescent cells [57,58]. A recent metabolomic analysis of cultured doxorubicin-treated breast cancer cells revealed that tricarboxylic acid cycle, pentose phosphate pathway, and nucleotide synthesis pathways were significantly upregulated, whereas fatty acid synthesis was reduced [59].

Altered mitochondrial function appears essential in mediating the senescence phenotype. RNA sequencing analysis has shown that a great number of senescence-associated changes involve the mitochondria and Akt (protein kinsase B), ATM (ataxia-telangiectasia mutated) and mTORC1 phosphorylation have been shown to link DDR with mitochondrial biogenesis [60]. Morphological changes in mitochondria are also seen in senescent cells [20,61]. In addition, impaired mitophagy seems to explain accumulation of dysfunctional mitochondria (senescence-associated mitochondrial dysfunction—SAMD) seen in cellular senescence [62,63]. Although mitochondria are not the sole source [63], they are a major generator of ROS, important for both cellular signaling and SASP [47,64,65,66].

Several epigenetic modifications are also common in cellular senescence. Defects in pericentric heterochromatic silencing at mammalian centromeres, normally regulated by SIRT6 (sirtuin 6) have been described [67]. SIRT6 belongs to the sirtuin protein family, whose function has been linked to longevity [68]. Micro-RNAs, a subclass of regulatory, non-coding RNAs that participate in regulation of cellular senescence may also be epigenetically modified [69]. Chromatic alterations such as senescence-associated heterochromatin formation (SAHF) may accompany cellular senescence in some settings with deactivation of proliferation-related genes [70,71,72,73]. Cellular senescence may also lead to changes in the organization of nuclear lamina and down regulation of lamin B1 with implications for nuclear morphology and gene expression [74]. 

Finally, senescent cells also undergo morphological changes. Cells become larger and flattened out and acquire an irregular shape. These alterations are more prominent in vitro than in vivo and appear to be caused by cytoskeletal rearrangements [75,76] and changes in cell membrane composition [77]. Senescent cells exhibit increased unfolded protein response (UPR), indicative of endoplasmic reticulum (ER) stress [76,77]. The ATF6α pathway of UPR appears to be responsible for both increasing ER size associated with ER stress and regulating the shape and size of senescent cells [76,77].

## 4. Markers of Cellular Senescence

To better understand the role of senescent cells in physiological and pathological conditions, it is essential to be able to detect them in vitro and in vivo [15]. So far, research on cellular senescence has been hindered by our lack a universal, specific and widely applicable marker of cellular senescence Here, we discuss the most commonly used markers of senescence:

- **p16^INK4a^**: This member of the INK4a family is a cyclin D-dependent kinase CDK4 and CDK6 inhibitor, which prevents the phosphorylation of the retinoblastoma protein (Rb), therefore leading to suspension of the cell cycle before the S-phase [78,79]. Increased levels of p16^INK4a^ have been documented in aged and stressed tissues, compared to younger, healthy tissues, whereas the removal of p16^INK4a^-expressing senescent cells in mice prevented or delayed tissue dysfunction and age-related disorders [38,80]. This evidence has established p16^INK4a^ as a widely-accepted marker of ageing and cellular senescence [81,82]. Limitations include poor detection of p16^INK4a^ in mice by the currently available antibodies using immunohistochemistry [79], as well as some situations, where p16^INK4a^ levels are increased, in the absence of other signs of cellular senescence [81,83,84].
- **p21^CIP1/WAF1/SDI1^**: p21 is a member of the second group of CDK inhibitors, namely the CIP/KIP (CDK interacting protein/kinase inhibitory protein) family and can inhibit a variety of CDKs [85]. Apart from its role in cellular senescence, it is a key mediator in several biological functions, including cell cycle arrest, cell death, DNA repair processes and even reprogramming of differentiated somatic cells into pluripotent stem cells [86]. In the context of senescence, stress-induced p53 activates p21 in order to trigger cell cycle arrest [39]. Although both p21 and p16^INK4a^ upregulation lead to cell cycle arrest, they act through different pathways and have distinct roles in the induction and progression of cellular senescence [36].
- **SA-β-gal**: The activity of β-galactosidase detectable at pH 6.0, which is measured using in situ staining with the chromogenic substrate X-gal [senescence-associated β-galactosidase activity (SA-β-gal)] is today the most widely used biomarker for detecting senescent cells [79,87]. The lysosomal enzyme β-galactosidase encoded by the GLB1 (galactosidase beta 1) gene, is the source of SA-β-gal activity and it can, therefore, be elevated in any situation with increased lysosome number or activity [54,88]. It has also been reported that certain cell culture conditions can increase the level of SA-β-gal, giving a false positive result [88,89]. Another drawback of the SA-β-gal assay is that it can only be used on fresh or frozen tissues and not on formalin-fixed paraffin-embedded archival tissue samples, which significantly limits its spectrum of application [15,57].
- **Lipofuscin:** Intracellular lipofuscin aggregates consist of oxidized protein and lipid degradation residues and metal cations that cannot be degraded by lysosomal enzymes. Lipofuscin accumulates with age and its accumulation is a documented hallmark of senescent cells [90,91,92]. GL-13 (SenTraGor^TM^) is a biotinylated chemical compound derived from Sudan Black-B that specifically and strongly binds to lipofuscin [57,58]. Its ability to detect lipofuscin, not only on fresh tissues, but also on formalin-fixed paraffin-embedded archival samples and biological fluids gives a new perspective in the field of senescence markers [57,58,90,93].

The fact that senescence can be induced by different stimuli and is mediated by several diverse mechanisms, as well as the drawbacks of each senescence marker, has led most researchers to abandon the single marker approach and rather utilize a combination of different biomarkers. However, to date, there is no consensus on the optimal combination of markers to detect senescence in vivo and in vitro [23,28,79].

## 5. Cellular Senescence and Its Putative Role in Neurodegeneration

A primary causative role of cellular senescence in neurodegenerative disease is highly unlikely given the great diversity which characterizes ageing-related neurodegenerative pathologies. However, cellular senescence may still substantially contribute to the pathogenesis of neurodegenerative disease and thereby determine disease susceptibility, age at disease presentation and rate of progression. Three mechanisms could explain the putative role of cellular senescence in neurodegeneration:

- **Promotion of chronic inflammation:** Senescence-associated secretory phenotype (SASP) converts senescent cells into continuous sources of pro-inflammatory mediators, reactive oxygen species and metalloproteinases [43]. Senescent cells may sustain a proinflammatory milieu, which can be damaging for neighboring cells or “contaminating” in the sense of converting neighboring cells to senescent ones in a paracrine manner [44,94,95]. Interleukin-6, a typical SASP mediator, is upregulated in the aged brain and in AD [96,97,98] and its overexpression has been shown to drive neurodegeneration in vivo [99]. The ageing brain has higher background levels of low-grade inflammation primarily in the form of dystrophic microglia and increased levels pro-inflammatory cytokines and other mediators, a state known as inflammaging [100,101,102]. SASP-related mediators from increased numbers of senescent cells may be what underlies inflammaging [103,104]. There are many links between inflammaging and AD and PD pathologies [105,106], which suggest that SASP may contribute to the pathogenesis of neurodegeneration and may determine disease susceptibility or aggravate the course of the disease.
- **Exhaustion of the regenerative capacities of the nervous system:** There is evidence of neurogenesis from adult neural stem cells deriving from the subventricular zone (SVZ) and the subgranular zone (SGZ) of the hippocampal dentate gyrus, that can give rise to neurons, oligodendrocytes and astrocytes [107,108]. Ageing has been shown to significantly reduce adult hippocampal neurogenesis [109]. Cell cycle arrest of adult progenitor cells in the context of cellular senescence may reduce the regenerative capacities of the CNS. This notion is supported by recent in vivo evidence from the BUBR1 progeroid mouse model in which adult progenitor proliferation was impaired in the SGZ and SVZ in an age-dependent manner [110]. Although the role of adult neurogenesis in AD remains contentious, studies from animal models indicate that ablation of adult neurogenesis exacerbates memory deficits and upregulates hyperphosphorylated tau [111], whereas implantation of human neural stem cells alleviates memory deficits and AD pathology [112].
  Furthermore, oligodendrocyte progenitor cells (OPCs) are a population of adult stem cells responsible for mediating CNS myelin repair in demyelinating conditions such as MS [113,114]. However, despite remyelination being very efficient at the early stages of the disease, this process gradually fails over time [115,116]. Evidence from MS and its animal models suggests that remyelination can protect demyelinated axons and even correlates with greater age at death, whereas chronically demyelinated axons are prone to degeneration [117,118]. Inability to replenish adult progenitor cells due to cellular senescence could render the CNS susceptible to neurodegeneration.
- **Loss of function:** The functional state of senescent cells has not been fully elucidated. However, cell-cycle arrest, changes in gene expression and phenotypic changes that accompany cellular senescence constitute serious restrictions in the functionality of different cell types [119]. The number of senescent cells increases with age [30]. At the same time it must be noted that ageing is associated with loss of brain cells to an extent which may amount to up to 0.4% of brain volume, annually [120]. The processes that lead to loss of brain cells with normal ageing are unclear. Both apoptotic and senescent cells can be cleared by the immune system in a highly regulated manner [35]. Thus, brain volume loss may at least partly be due to immune-mediated clearance of senescent cells. It is also conceivable, that when the number of dysfunctional senescent cells exceeds a certain threshold in a brain with reduced reserves due to age-related cell loss, nervous tissue function is likely to become compromised. Senescent cell accumulation may occur preferentially in some brain regions e.g., substantia nigra, that are more susceptible to particular stressors, which could explain the ensuing functional deficits.
- **Cerebral hypoperfusion and blood-brain barrier (BBB) dysfunction:** Cerebral function depends on an adequate blood supply and an intact BBB, which is crucial for maintaining homeostasis of brain microenvironment and protecting the parenchyma from pathogens, circulating immune cells, ionic changes and toxic metabolites [121]. There is evidence of an age-related decline in cerebral microvascular structure [122] and vascular pathology has been shown to accompany age-related cognitive impairment and neurodegeneration [123]. BBB leakiness is seen both with normal ageing and AD [124,125]. In a model of AD in transgenic mice BBB permeability increase even preceded neuritic plaque formation [126] and in a neuropathological study, the ApoE4 allele, which is a major risk factor for developing AD, was associated with greater likelihood of BBB disruption [127]. Vascular cells and specifically endothelial cells and pericytes have been shown to undergo senescence in vitro and in vivo [128]. Accumulation of senescent endothelial cells is associated with impaired tight junction structure and compromised blood-brain barrier integrity [129] and it is linked to Sirt1 downregulation in senescent endothelial cells [130]. Although senescence has not been studied in the cellular components of the choroid plexus, it is known to undergo several age and disease-related structural and functional alterations [131,132]. The choroid plexus produces CSF and forms an interface between blood and CSF. It secretes trophic factors, such as epidermal growth factor (EGF) and fibroblast growth factor-2 (FGF-2) and may be a route for trafficking lymphocytes to and from the CNS with roles in immune surveillance and neuroinflammation [133,134]. In addition, a shift towards an interferon I-dependent expression profile is seen with ageing in human and mouse choroid plexus, which may adversely affect cognitive function and hippocampal neurogenesis [135]. It is conceivable that compromised cerebrovascular perfusion and altered function of the BBB and/or choroid plexus may adversely affect neuronal and glial survival.

## 6. Evidence of Cellular Senescence in CNS Cell Types

### 6.1. Astrocytes

The astrocyte is the most abundant cell type of the CNS with a prominent role in the complex functions of the healthy CNS, as well as in various pathologies [136]. Over the last few years, evidence concerning astrocyte senescence has started to emerge. It has been reported that cultured rat astrocytes show characteristics of senescence, such as increased SA-β-gal staining, robust ROS production and decreased mitochondrial activity, resulting in the loss of their ability to maintain neurons and therefore exerting detrimental effects in the aging brain [137,138]. 

SASP appears to be another important component of astrocyte senescence [139]. Glutathione depletion in human astrocyte cultures activated SASP-associated pathways (NF-κB and p38MAPK) and triggered secretion of IL-6 [140]. Other studies also showed that cultured astrocytes of human and rodent origin can undergo both stress-induced and replicative senescence, which is, interestingly, telomere-independent. They are characterized by an enlarged and flattened morphology and increased levels of p53, p21^CIP1^, p16^INK4a^ and SA-β-gal, as well as the formation of SAHF [141,142]. 

Several substances and environmental toxins have been associated with astrocyte senescence. The dioxin TCDD can induce premature senescence in rodent astrocytes through activation of the WNT/β-catenin signaling pathway and ROS production and is characterized by increased levels of senescence markers, such as SA-β-gal, p16 and p21 [143]. Ammonia has also been shown to trigger cellular senescence in cultured rat astrocytes, mediated by ROS and p38MAPK activation leading to growth arrest and elevated SA-β-gal and p21 levels [144]. Paraquat can induce astrocyte senescence and SASP in vitro, characterized by elevated levels of SA-β-gal and p16^INK4a^, secretion of IL-6 and increased number of 53BP1 foci [145]. These data provide a mechanistic link between environmental factors, cellular senescence and the risk of neurodegenerative disease [146]. 

A recent study by Crowe et al. (2016) reported that oxidative stress-induced senescence can cause several transcriptomic changes on human astrocytes. More specifically, genes associated with the development and differentiation of the nervous system, as well as cell cycle genes were downregulated, whereas genes associated with inflammation, extracellular remodeling and apoptosis resistance were upregulated [147]. Aβ has been shown to trigger astrocyte senescence with increased production of IL-6 regulated by p38MAPK [148], which corroborates a potential role of astrocytic senescence in AD pathology. In line with these results, Hou et al. also reported that SASP is expressed in senescent astrocytes and regulated by p38MAPK in a NF-κB-dependent manner [149], while Mombach et al. designed a logical model, where p38MAPK has a central role in the regulation of astrocyte senescence and SASP, in response to DNA damage [150]. Finally, prematurely aged BUBR1 mutant mice display alterations in gliosis from activated astrocytes, providing in vivo evidence of a link between accelerated cellular senescence and astrocytic dysfunction [151]. 

### 6.2. Microglia

Microglial cells are of mesenchymal origin and are the main representative of innate immune response in the CNS [152]. Microglial cells have been shown to undergo senescence with typical features. Cultured rat microglial cells have been reported to undergo replicative senescence due to telomere shortening [153] and the same finding was later reported for microglial cells from AD patients [154]. Liposaccharide treatment of BV2 microglial cells in culture led to the development of a senescence-like phenotype with growth arrest, SA-β-Gal upregulation and SAHF [155]. With ageing, microglial cells exhibit dystrophic changes, which are thought to be distinct from their typical reactive morphology. This dystrophic microglial phenotype is also associated with functional changes, it is more abundant in neurodegenerative conditions such as AD and may even precede the onset of neurodegeneration, indicating that there may be a causal relationship between microglial senescence and neurodegeneration [100,156,157]. In addition, an RNA sequencing study of age-related transcriptional changes in astrocytes revealed that in the aged mouse brain astrocytes acquire a pro-inflammatory reactive phenotype in response to induction by microglial cells. Nonetheless, this study did not examine any senescence markers that would allow us to attribute this age-related pro-inflammatory state of microglia and astrocytes to cellular senescence and SASP [158].

### 6.3. Oligodendrocytes

Oligodendrocytes are terminally differentiated post-mitotic cells that form the myelin sheaths of myelinated axons. They are known to be extremely vulnerable to oxidative stress [159]. Evidence of oxidative DNA damage and upregulated SA-β-Gal suggest that oligodendrocytes may undergo stress-associated cellular senescence in ageing individuals [160]. Neuroimaging and neuropathological data indicate that there is myelin damage in the white matter associated with ageing and AD [161,162,163], which could at least be partially explained by oligodendrocyte senescence [164].

### 6.4. Oligodendrocyte Progenitor Cells

Oligodendrocyte progenitor cells (OPCs) are a population of adult progenitors which constitute approximately 3–10% of glial cells [165]. Under some circumstances they are capable of undergoing asymmetric division and mediate remyelination by differentiating into myelinating oligodendrocytes, a process highly relevant to myelin repair in multiple sclerosis [113]. Although OPCs do not undergo replicative senescence [166], there is in vitro evidence that under some circumstances they enter a senescence-like state. OPC senescence is induced by the esophageal cancer-related gene 4 (Ecrg4) and is characterized by cell cycle arrest and increased expression of SA-β-Gal [167]. Interestingly, Ecrg4 exhibits increased expression in OPCs and neural stem cells (NSCs) in the aged mouse brain. It has been noted that spontaneous remyelination in MS fails with ageing [118,168,169]. In addition, BUBR1 insufficiency, which causes a state of accelerated senescence impairs adult OPC proliferation in vivo [170]. Oligodendrocytes and oligodendrocyte precursor cells (OPCs) dysfunction, as well as myelin breakdown have been suggested to also play an important role in the pathogenesis and progression of AD, although the exact mechanisms remain unclear [164,171].

### 6.5. Neurons

Although neurons are post-mitotic and don’t fit the strict definition of cellular senescence, several lines of evidence suggest that even mature post-mitotic neurons develop a senescence-like phenotype. Neurons of aged mice accumulate increased amounts of double strand DNA breaks, SA-β-Gal and proinflammmatory cytokines [172]. About 20–80% of mature neurons of aged mice exhibit a senescence-like phenotype with increased levels of DNA damage, heterochromatinization, SA-β-Gal activity, p38MAPK activation and production of SASP-related mediators including ROS and IL-6 [173]. Interestingly, this senescence-like phenotype was aggravated by a genetic background of dysfunctional telomeres (terc KO mice) and rescued by a CDKN1A KO background, indicating that the senescence-like phenotype is p21-mediated in aged murine neurons [173]. The demonstration of granular cytoplasmic lipofuscin deposits with ageing [174] supports the notion that human neurons may also acquire an ageing-related senescence-like phenotype. There is little data regarding the functional activity of these senescent-like neurons. Nevertheless, neurons from nuclei of the sleep-wake cycle seem to be particularly prone to lipofuscin accumulation with ageing and those lipofuscin positive neurons exhibited poorer dendritic arborization and decreased neurotransmitter production, indicative of functional compromise [175]. In addition, neurons deriving from reprogrammed fibroblasts from patients with Rett syndrome, a neurodegenerative condition due to a MECP loss of function mutation, exhibit evidence of double strand DNA damage and p53-mediated SASP, providing in vitro evidence of a link between cellular senescence and neurodegenerative disease in humans [176]. 

### 6.6. Neural Stem Cells (NSCs)

The therapeutic potential of NSCs in AD has been under thorough investigation in the last few years [177]. Meanwhile, accumulating evidence suggests that these cells are also prone to senescence. NSCs may undergo senescence in vitro in response to various stressors [178]. Specifically, after long-term incubation with Aβ oligomers, cultured NSCs have been reported to exhibit characteristics of senescence, such as enlarged and flattened morphology, increased levels of SA-β-gal and p16 and decreased level of pRb, a response mediated by the p38MAPK pathway [179,180]. These senescent NSCs have also been observed in the dentate gyrus of the APP/PS1 transgenic mouse AD model [179]. NSCs exhibit features of cellular senescence such as telomere shortening, and ROS production with ageing [181,182]. Furthermore, there is evidence from the BUBR1 KO mouse that accelerated cellular senescence impairs adult neurogenesis in vivo [110].

## 7. Cellular Senescence in Alzheimer’s Disease, Parkinson’s Disease and Multiple Sclerosis

### 7.1. Alzheimer’s Disease

Cognitive decline in AD is associated with the disseminated formation of extracellular amyloid plaques, intracellular neurofibrillary tangles comprising of hyperphosphorylated tau proteins, as well as neuronal and synaptic loss [183]. A plethora of evidence links cellular senescence with AD. Aβ42 oligomers are reported to trigger the senescent phenotype in in vitro studies with mouse neural stem cells, leading to increased numbers of SA-β-Gal positive cells [179]. Several in vivo studies in mouse models of AD corroborate these findings [179]. Increased level of SA-β-Gal was also found in plasma samples from AD patients, compared to controls [184,185]. However, SA-β-Gal was significantly decreased in monocytes and lymphocytes from AD patients compared to controls, a finding attributed to the up-regulation of miR-128 [186].

Cumulative evidence suggests that aberrant cell cycle re-entry of the terminally differentiated post-mitotic neurons may play a critical role in the pathogenesis of AD, a theory that is supported by the re-expression of several cell-cycle regulating proteins in vulnerable neurons [187,188,189,190]. More specifically, the cyclin-dependent kinase inhibitor p21^CIP1^, appears to be a critical mediator of cell-cycle dysregulation in AD [191]. However, the evidence remains inconclusive, as a number of studies have reported increased levels in the brains of AD patients compared to controls [192,193], while others have found no significant differences [194]. Interesting are also the results from AD and tauopathy mouse models [195,196], as well as from studies of peripheral blood lymphocytes and monocytes of AD patients [197,198]. The levels of p16^INK4a^ have been reported to be elevated in neurons from AD patients [194,198,199,200], as well as in neurons from AD mouse models [195]. Increased levels of p53, a key mediator of cellular senescence and apoptosis, have been reported in different brain regions and in lymphocytes from AD patients [192,201,202,203], as well as in neurons of mouse models of AD [204].

Increased p38MAPK activity has been reported in AD brains and lymphocytes [205,206,207,208], as well as in the cortex of a mouse model of AD [209]. Since p38MAPK is a major regulator of SASP [209], it is not surprising that a number of key components of SASP appear to be up-regulated in AD [210,211]. Most notably, IL-6, IL-1β, TGF-β and TNF-α levels have been reported to be elevated in AD brain tissue [96,97,212,213,214], as well as in AD patients’ CSF and serum [215,216,217,218,219,220,221,222], while increased levels of metaloproteinases MMP-1, MMP-3 and MMP-10 have also been reported in AD [223,224,225,226].

Epigenetic modifications appear to play an important role in the pathogenesis of the disease, as differences in overall methylation have been observed in AD-affected brain regions and abnormal DNA methylation patterns have been reported in several genes associated with AD [227,228,229]. Moreover, elevated phosphorylated histone γH2AX (H2A histone family member X) levels have been reported in the hippocampus and lymphocytes from AD patients, indicating an active DNA damage response [230,231].

Several lines of evidence suggest that deficits in autophagy and lysosomal dysfunction contribute to the etiology and progression of neurodegenerative diseases and especially AD [232,233]. This is supported by a number of studies reporting dysregulation in many autophagic/lysosomal pathways in the context of AD [234,235], while the vast majority of AD-associated genes appears to be related to these same pathways [232]. A recent study attempted to shed light on the interplay between autophagic/lysosomal impairment and mitochondrial dysfunction and their relation to stress-induced premature senescence (SIPS) [236]. All aspects of mitochondrial function have been reported to be impaired in AD [237], including aberrant mitochondrial dynamics and structure [238] and increased oxidative stress, which is already present in the very early stage of the disease and precedes the major pathologic hallmarks, such as senile plaques and neurofibrillary tangles [132,193]. Therefore, mitochondria and lysosomes appear to have a critical role in the progression of SIPS [239], although further research is needed to elucidate their exact contribution to AD and senescence. 

Besides neurons, all different cell types that are involved in AD pathology have been reported to undergo senescence. Astrocytes are key players in the initiation and progression of the disease and can have both beneficial and detrimental effects, depending on different factors [240]. Aβ oligomers can induce senescence in human astrocytes and through the activation of p38MAPK pathway lead to the production of SASP components, such as IL-6 and MMP-1 [128]. Furthermore, increased levels of γH2AX have been found in astrocytes from AD hippocampal samples [241]. Microglia has long been implicated in the pathogenesis of AD, although the exact underlying mechanisms remain elusive [242,243]. Cultured microglial cells from AD patients have been reported to undergo replicative senescence due to telomere shortening [153]. Moreover, neuropathological features of AD have been associated with dystrophic microglial cells that exhibit morphological changes indicative of senescence [157]. A recent study reported that in vitro aged microglia from rats [244], after treatment with Aβ oligomers acquire a senescent phenotype, characterized by increased levels of SA-β-gal, IL-1β, TNF-α and MMP-2 [245]. Finally, an association between telomere shortening and AD has been suggested [246,247,248]. However, a large community-based longitudinal study reported no difference in the telomere length between incident pure AD patients and cognitively healthy individuals [249]. More studies are needed to shed light on the plausible connection between telomere length and AD.

### 7.2. Parkinson’s Disease

PD pathology is mainly characterized by loss of neurons from the substantia nigra pars compacta in association with the accumulation of ubiquitinated alpha synuclein and other proteins in cytoplasmic inclusions (Lewy bodies) and thread-like proteinaceous inclusions within neurites (Lewy neuritis). However, Lewy bodies are also seen in the cerebral cortex, brainstem nuclei, limbic system, sympathetic ganglia, nucleus basalis of Meinert and myenteric plexus [4]. A great deal of data supports a role of cellular senescence in the pathogenesis of PD. The expression of cell-cycle genes has been found upregulated in PD. Specifically, p16^INK4a^ mRNA levels were elevated in PD brain samples compared to controls [145]. Increased pRb, another important regulator of cell-cycle progression, was reported in the cytoplasm of neurons in the substantia nigra of PD patients compared to age-matched controls [234]. The same study showed that the serine 795 phosphorylated, inactive form of pRb (ppRb), had a distinct distribution pattern in PD cases [250]. Another study reported increased levels of the E2F-1 transcription factor in dopaminergic neurons in the substantia nigra of PD patients and suggested that the pRb/E2F-1 pathway is activated in these neurons which can lead to apoptosis [251]. Increased levels of SA-β-gal have also been found in the CSF from PD patients compared to healthy controls [252].

Several SASP-related factors have been found upregulated in PD. IL-1β levels have been reported to be elevated in the CSF [218], serum [222] and dopaminergic regions of the striatum from patients with PD compared to controls [253]. Increased levels of IL-6 in PD patients’ serum have been reported in a number of studies [254,255,256], while IL-6 levels have also been associated with disease severity [257]. IL-6 levels have also been found elevated in the striatal dopaminergic region [253], as well as the CSF from PD patients compared to controls [218]. Elevated TNF-α levels have been reported in the striatum and the CSF of PD patients compared to controls [258], while MMP-3 was found to co-localize with α-synuclein in the Lewy bodies in PD patients’ brains [259]. However, it is not clear whether the elevated levels of these cytokines can be attributed to SASP in PD or they are merely part of a separate neuroinflammatory process, which is an established part of the pathophysiology of the disease [260].

Several lines of evidence indicate that mitochondrial dysfunction plays a central role in the pathophysiology of PD. Different mutations in the genes involved in familial PD are associated with pathways of mitochondrial dysfunction, while some of these compromised pathways have been established as important factors in the pathophysiology of sporadic PD [261]. Autophagic/lysosomal dysfunction are also thought to have a key role in the pathogenesis of the disease, with many PD mutations being associated with defects in these pathways [262,263]. Interestingly, a number of key mutations are involved both in mitochondrial and autophagic/lysosomal dysfunction pathways, revealing a compelling crosstalk that lies in the center of the pathophysiology of PD [264].

A recent study by Chinta et al. found increased numbers of senescent astrocytes in substantia nigra tissue samples from PD patients, as compared to controls [145]. The same study also reported that paraquat, an herbicide that has been strongly associated with the development of sporadic PD, was able to induce senescence in human astrocytes [145]. 

The evidence concerning telomere length in PD remain inconclusive, [246] with a number of different studies reporting contradictory results [265,266,267,268,269,270]. A meta-analysis by Forero et al., incorporating all these studies, showed that there is no difference in telomere length between PD patients and age-matched controls [270].

### 7.3. Multiple Sclerosis

Multiple sclerosis (MS) is a chronic, immune mediated disease characterized by inflammatory demyelination, astrogliosis, neuronal and axonal loss involving the brain and spinal cord [271]. Its aetiology remains unclear but genetic and environmental factors are thought to influence the likelihood of developing the disease [272]. The majority of MS patients follow an initial course with relapses followed by some degree of remission called relapsing-remitting MS (RR-MS). Relapses in RR-MS are driven by inflammation which can be visualized as new focal inflammatory demyelinating lesions with magnetic resonance imaging (MRI) techniques. Several immunomodulatory and immunosuppressive disease-modifying treatments are currently available with moderate to high efficacy in tackling inflammation in RR-MS [273]. Nevertheless, after variable time RR-MS gradually transforms into secondary progressive MS (SP-MS), a phase with progressive build-up of disability. In the secondary progressive phase of MS (SP-MS) new focal inflammatory demyelinating lesion formation is rare, and the pathological correlate of disability progression is neuroaxonal loss driven by neurodegenerative mechanisms [274,275,276]. The pathogenesis of ongoing neuroaxonal loss and the time of shifting from the relapsing to the secondary progressive phase of the disease are poorly understood. Epidemiological evidence suggests that age is the most important determinant for the transition to the progressive phase of MS [11]. Several immunomodulatory and immunosuppressive therapies have failed in the progressive forms of MS. Licensed therapeutic options for preventing disease progression in SP-MS are lacking. 

Oxidative damage and mitochondrial dysfunction are key features of MS pathology [277,278,279,280]. Cellular senescence is an age-dependent process known to be accelerated by oxidative stress and chronic inflammation [14,20]. Random irreparable ROS-mediated damage to the DNA of cells and mitochondrial dysfunction are strong inducers of cellular senescence [14]. We postulate that accelerated accumulation of senescent cells above a certain threshold may determine the shift to the secondary progressive phase of MS and that neurodegeneration in progressive MS is driven by senescence-associated loss of function. Furthermore, the so-called “compartmentalized within the blood-brain barrier (BBB)” inflammation [276], which is resistant to our immunomodulatory strategies, may represent the SASP-associated low burning inflammation. In line with our hypothesis, currently used immunomodulatory and immunosuppressive treatments are modestly or not effective in the secondary progressive phase of the disease but may delay the onset of the secondary progressive phase when used early in the inflammatory relapsing phase [281], probably due to their efficacy in preventing new inflammatory demyelinating lesion formation and the oxidative DNA damage associated with it. 

**Figure 1:**
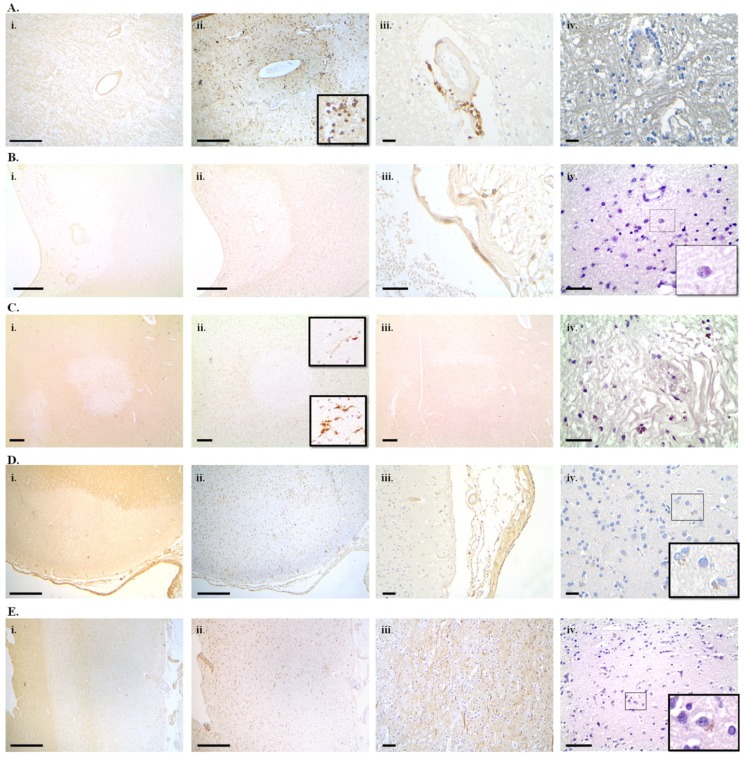
Lipofuscin accumulation as a marker of cellular senescence in multiple sclerosis lesions. Demyelinated lesions were identified with myelin basic protein (MBP) immunohistochemistry and were staged according to Trapp et al. (1998) [287] as acute active, chronic active or chronic inactive using human leukocyte antigen-DR isotype (HLA-DR) immunohistochemistry on serial sections from paraffin embedded postmortem tissue blocks. Lipofuscin was detected with the GL13 hybrid histochemistry-immunohistochemistry method [58]. Acute active demyelinated white matter lesion with MBP staining showing ongoing perivascular demyelination in subcortical white matter from the parietal lobe of a 73-year-old secondary progressive multiple sclerosis (SP-MS) patient (MS51) (**A**(**i**))**.** Infiltration with HLA-DR^+^ cells with macrophage morphology throughout the demyelinated parenchyma (HLA DR immunohistochemistry) (**A**(**ii**)). Perivascular infiltration with CD8^+^ lymphocytes (CD8 immunohistochemistry) (**A**(**iii**)). GL13 staining in acute active lesions showed lipofuscin^+^ cells. Although many of them were perivascularly localized, some were not, suggesting that at least some of them maybe glial cells rather than inflammatory cells (**A**(**iv**)). Chronic actively demyelinating perivenentricular white matter lesion with a fully demyelinated lesion center (lack of MBP immunoreactivity) from a 74-year-old female MS patient (MS265) (**B**(**i**)). Typically, HLA-DR immunohistochemistry of serial sections exhibited a border infiltrated by numerous macrophages whereas the lesion centre is infiltrated by ramified microglia (**B**(**ii**)). Few CD8^+^ lymphocytes are present perivascularly (CD8 immunohistochemistry) (**B**(**iii**)). Lipofuscin^+^ cells with granular staining were found in the macrophage infiltrated lesion border (**B**(**iv**)). Chronic inactive subcortical white matter demyelinated lesion (lack of MBP immunoreactivity with a well demarcated border) from the left parietal lobe of a 71-year-old female SP-MS patient (MS33) (**C**(**i**))**.** Ramified microglial morphology throughout the demyelinated lesion area and lesion border (HLA-DR immunohistochemistry) typical of a chronic inactive lesion (**C**(**ii**)). Decreased axonal density in the demyelinated lesion seen with 200 KDa neurofilament immunohistochemistry (**C**(**iii**)). Numerous parenchymal lipofuscin^+^ cells in the demyelinated white matter. Lack of HLA-DR^+^ macrophages from the chronic demyelinated lesion suggests that the lipofuscin^+^ cells are glial (**C**(**iv**)). Subpial cortical demyelination (lack of MBP immunoreactivity extending from the pial surface into the deeper cortical layers from the parietal cortex of a 71-year-old female SP-MS patient (MS33) (**D**(**i**)). HLA-DR^+^ ramified microglia in the demyelinated cortical lesion (**D**(**ii**)) and few CD8^+^ lymphocytes infiltrating the adjacent pia matter (**D**(**iii**)). Lipofuscin^+^ cells mostly with neuronal morphology (inset) throughout the demyelinated cortex (**D**(**iv**)). Normal appearing cortex with intact appearing cortical myelin (MBP immunohistochemistry) from the left parietal lobe of a 71-year-old female SP-MS patient (MS33) (**E**(**i**)), HLA-DR immunoreactivity revealing quiescent ramified microglia (**E**(**ii**)) and normal-appearing axonal staining with 200 kDa neurofilament immunohistochemistry on a serial section (**E**(**iii**)). GL13 staining showed numerous lipofuscin^+^ cells mostly with neuronal morphology (**E**(**iv**)). Scale bars represent 500 μm (**A**(**i**),**A**(**ii**),**B**(**i**),**B**(**ii**),**C**(**i**),**C**(**ii**),**C**(**iii**),**D**(**i**),**D**(**ii**),**E**(**i**),**E**(**ii**)), 50 μm (**D**(**iii**),**E**(**iii**)) or 25 μm (**A**(**iii**),**A**(**iv**), **B**(**iii**), **B**(**iv**), **C**(**iv**), **D**(**iv**), **E**(**iv**)).

In the cuprizone-induced demyelination model of multiple sclerosis, increased numbers of senescence-associated β-galactosidase positive senescent glial cells were detected in the chronically demyelinated corpus callosum. This finding was confirmed with GL13 lipofuscin histochemistry. Correlation analysis revealed a significant association between the number of senescent cells and the extent of demyelination and motor performance, indicating a link between chronic demyelination and senescent glial cell load and between the senescent glial cell load and loss of function [282]. Using GL13 histochemistry as a marker for cellular senescence we detected lipofuscin^+^ glial cells in acute active (Figure 1A iv) and chronic active demyelinated white matter lesions from SP-MS cases (Figure 1B iv). Lipofuscin positive senescent cells were sparse in chronic inactive demyelinated lesions (Figure 1C iv). No lipofuscin^+^ glial cells were detected in the normal appearing white matter (NAWM) (data not shown). Cortical demyelination is common and extensive, particularly in the progressive stages of MS [283]. The extent of cortical demyelination has been shown to correlate with disability, cognitive impairment and the likelihood of developing seizures [284]. The most abundant type is the subpial cortical demyelinated lesion which extends from the pial surface into the deeper cortical layers. A gradient of neuronal loss greater at the most superficial layers I and II and lesser at deeper layers V and VI has been described [285]. Yet, cortical neuronal and synaptic loss have been demonstrated in the absence of demyelination [286]. GL13 histochemistry of subpial demyelinated cortical lesions (Figure 1D iv) and normal appearing cortex revealed granular lipofuscin deposits in numerous neurons (Figure 1E iv), indicating that neurons in SP-MS exhibit a senescence-like phenotype. Evidence of lipofuscin accumulation in glial cells and neurons in grey and white matter demyelinated lesions in SP-MS corroborate the hypothesis of cellular senescence playing a pathogenetic role in progressive MS. Nevertheless, these findings merit further quantitative investigation in order to differentiate the effects of ageing from those of MS and to potentially associate the extent of cellular senescence with other pathological features and clinical parameters.

## 8. Cellular Senescence as a Therapeutic Target

Currently, there are no available neuroprotective treatments that can effectively modify the disease course and prevent disease progression for AD or PD. Several attempts at targeting Aβ amyloid in AD have failed [288,289]. Interestingly, the Aβ plaques may be found in 35% of cognitively healthy individuals above the age of 60 [290] and the Aβ load, which has been our main target, correlates better with age than disease severity in AD [291,292,293], casting doubt on the amyloid hypothesis. Similarly, therapies in PD aim to substitute dopamine and restore the dopaminergic system deficit, with no effect on neuronal cell loss and consequently on disease progression. In MS only siponimod, an S1P_1/_S1P_5_ receptor modulator, which is not currently licensed, has shown a modest effect in a phase III trial (EXPAND) in secondary- progressive MS, preventing disability progression by 21% in two years [294]. Therefore, there is an urgent need for neuroprotective treatments for neurodegenerative disease. Investigation into new treatment approaches may require a paradigm shift in our view of the pathogenetic mechanisms of neurodegeneration. 

Several lines of evidence implicate cellular senescence in the pathogenesis of neurodegenerative disease. Targeting cellular senescence as a therapeutic strategy is promising yet still at an embryonic stage. There is evidence of a beneficial effect of both pro-senescent and anti-senescent approaches, depending on context. A pro-senescent effect may be desirable in treating cancer [295,296,297], renal, liver and cutaneous fibrosis [298,299,300,301]. An anti-senescent treatment approach may be beneficial in neurodegenerative disease. An anti-senescent or senotherapeutic approach may involve the selective death of senescent cells in order to reduce the burden of senescent cells on a tissue (senolysis) or modulate senescent cells (senomorphism) in a way that neutralizes the detrimental effects of senescent cells in a tissue i.e., by blocking the expression of SASP or particular mediators of SASP. 

Senolysis and/or senomorphism in neurodegenerative disease would aim at preventing cell loss and tissue destruction in order to ultimately prevent disease progression. Given that neurodegenerative diseases seem to have a long presymptomatic phase with pathological changes appearing several years before clinical presentation e.g., 50–60% of nigral neurons are already lost at PD diagnosis [302], a great effort is being made to diagnose neurodegenerative disease presymptomatically using different biomarkers. In the context of presymptomatic diagnosis, early senotherapeutic treatment could, in theory, even prevent the clinical presentation of neurodegenerative disease. So far, the most convincing senotherapeutic manipulation comes from a sophisticated experiment in genetically modified BubR1 progeroid mice. In this setting, P16^INK4A^ senescent cells were eliminated by activation of an INK-ATTAC transgene by drug treatment. Lifelong elimination of p16^INK4A^ cells substantially delayed age-related disease, whereas late life elimination of p16^INK4A^ cells attenuated these age-related pathologies [38], supporting the notion that cellular senescence can be successfully exploited therapeutically. 

A number of compounds with senolytic or senomorphic actions have been examined in vitro and in vivo with notable results, summarized in Table 1. From our limited experience so far it is evident that, in most cases, the senescence-modifying action is not universal, but rather cell-type dependent, which greatly complicates the therapeutic landscape [303]. Many of the promising compounds with senolytic or senomorphic activity such as metformin or dasatinib are in use with different indications (diabetes mellitus type 2 and CML/ALL, respectively), which suggests that drug repurposing may facilitate our quest for efficacious senotherapeutics. Nevertheless, senotherapeutics have not been examined in models of neurodegeneration and supportive evidence remains weak and indirect, and sometimes even contradictory. For example, although some epidemiological studies supported a protective role for metformin, which crosses the BBB, in preventing cognitive decline in individuals with type 2 diabetes [304], another 12-year cohort study in patients with type 2 diabetes showed a two-fold increase in the risk of AD and PD in those who took metformin compared to those who didn’t [305]. The complex physiological and pathophysiological roles of cellular senescence, exerting both beneficial and detrimental effects according to setting, along with the cell-type specific variability in senescence triggers and senescence phenotypes, necessitates a cautious approach to avoid pitfalls when targeting a such a key biological process therapeutically. Furthermore, the relationship between senescence and immune response merits further elucidation. Naturally occurring immune-mediated clearance of senescent cells could be exploited therapeutically by developing medications that enhance it. The high specificity of immune responses could be employed to specifically target senescent cell types by developing senolytic vaccines. Cell surface markers of senescence [306] or even the intracellularly localized lipofuscin could potentially be used to prime the cellular or humoral immune response, directing it against senescent cells. Expansion of our knowledge of cellular senescence and its extensive investigation in numerous settings is warranted.

## 9. Conclusions and Future Perspectives

Senescent cells accumulate with ageing and progeroid models have provided in vivo experimental data of accelerated ageing-related degenerative pathologies. In addition, experimental senolysis ameliorated ageing-related pathologies [38]. Thus, cellular senescence meets the criteria for a potentially causal role in ageing-related disease. There is evidence of cellular senescence affecting astrocytes, microglia, oligodendrocyte progenitors and neural stem cells. A senescence-like phenotype has also been demonstrated in post-mitotic cells, which suggests that neurons and oligodendrocytes may also become senescent. Resident brain cells are either post-mitotic or slowly cycling. They are more likely to exhibit stress-induced premature senescence due to various stressors or insults than develop replicative senescence. However, evidence connecting cellular senescence with the mechanisms of neurodegeneration remains indirect and further investigation of the putative role of senescence in neurodegeneration is required. Unequivocal identification of senescent cells in vitro and in vivo is an important prerequisite to facilitate our understanding of cellular senescence and its role in different cell types. Detecting lipofuscin as a marker of cellular senescence using the GL13 compound, which not only detects lipofuscin in situ but also in biological fluids [93], is likely to boost our understanding of the senescence process. Casting light on CNS cell senescence and its role in neurodegeneration is essential to inform any practices that may be senescence- inducing e.g., using corticosteroids [368], beta-interferons [370] or DNA-damaging chemotherapeutics in MS, practices that may prove detrimental in the long run. Secondly, there is an urgent need for disease-modifying cures for neurodegenerative diseases. Cellular senescence may be a credible therapeutic target opening new therapeutic avenues for neurodegenerative disease and senotherapeutics may prove to be efficacious neuroprotectants.

## Figures and Tables

**Table 1:** Licensed and experimental compounds with senolytic, senomorphic or senescence-inducing action adapted from Myrianthopoulos et al., 2018 [303]. ALL: acute lymphocytic leukemia, CML: chronic myelogenous leukemia, HUVEC: human umbilical vein epithelial cells, JAK: Janus kinsase, MEF: mouse embryonic fibroblasts, MoA: mechanism of action, PPI: protein-protein interaction inhibitor.

Compound	MoA	Effect	Current Indication	Classification	References
Dasatinib (Sprycel)	Tyrosine kinase inhibitor, Inhibitor of Eph receptors	Reduced proliferation of senescent cells in vitro; Alleviated ageing phenotypes in treated animals	Philadelphia chromosome-CML and ALL	senolytic	[307,308,309,310]
Quercetin	Modulator of NF-κB, PI3K/Akt, estrogen receptor, mTOR, PIKδ kinase inhibitor, Potent antioxidant	Kills senescent human endothelial cells and murine bone marrow mesenchymal stem cells	experimental	senolytic	[311,312,313,314,315,316,317,318,319,320,321,322,323]
Navitoclax (ABT-263)	BCL-2 inhibitor (PPI)	Reduced survival of HUVEC, human lung fibroblasts and murine embryonic fibroblasts and mesenchymal stem cells in vitro	experimental	Senolytic	[324,325,326,327,328,329,330]
ABT-737	BCL-XL inhibitor (PPI)	Reduced viability of senescent cell in vitro and in vivo	experimental	senolytic	[331,332,333,334]
A1331852 and A1155463	BCL-XL inhibitor	Reduced viability of senescent cell in vitro.	experimental	senolytic	[335]
Fisetin	Interacts with: topoisomerases, cyclin-dependent kinases, NF-kB, PPAR, PARP1, PI3K/Akt/mTOR, antioxidant	Delay of age-related CNS complications in vivo	experimental	senomorphic	[335,336,337,338,339,340,341,342,343,344,345,346]
Piperlongumine	NF-kB modulator	Induces apoptosis in aged cells	experimental	senolytic	[347,348]
Geldanamycin	HSP90 inhibitor, down-regulation of PI3K/Akt	Induces death of senescent cells in vitro	experimental	senolytic	[349,350]
Tanespimycin (17-AAG)	HSP90 inhibitor, down-regulation of PI3K/Akt	Induces death of senescent cells in vitro	experimental	senolytic	[349,350]
Panobinostat (Farydak)	non-selective histone deacetylase inhibitor	Synergistic effect with taxol in inducing death of senescent cells, in vivo	multiple myeloma	senolytic	[351]
Apigenin and Kaempferol	Interference with the NF-kB p65 subunit and IkB	Inhibited components of SASP such as in IL-6, CXCL and GM-CSF in vivo.	experimental	senomorphics	[352]
Rapamycin (Rapamune)	mTOR kinase inhibitor	suppression of replicative senescence of rodent embryonic cells, lifespan extension in model systems	Lymphangio-myomatosis, coronary stent clot prevention, prevention of transplant rejection	senomorphic	[353,354,355,356]
Ruxolitinib (Jakafi)	JAK inhibitor	decreased systemic inflammation in aged animals and improved age-related dysfunctions and alleviating frailty	Polycytemia vera, Myelofibrosis	senomorphic	[357]
Metformin (Glucophage^TM^)	Inhibition of phosphorylation of IkB kinase	Increases lifespan, inhibits SASP, Prevents senescence in a disk degeneration model	Diabetes mellitus type 2	senolytic	[358,359,360,361]
Cortisol and corticosterone		Prevented senescence of human fibroblasts in vitro	Inflammation, allergy	senomorphic	[362]
Resveratrol and derivatives	SIRT1 and IkB inhibitor	Attenuates SASP in human fibroblasts in vitro	Dietary supplement	Senomorphic/senolytic/senescence modulator	[363,364,365]
Loperamide	Opioid receptor agonist and Ca++ channel blocker	Prevented senescence of primary MEFs in vitro	antidiarrheal	senomorphic	[349]
Niguldipine	Ca^++^ channel blocker, a1 adrenoreceptor antagonist	Prevented senescence of primary MEFs in vitro	experimental	senomorphic	[349]
Nutlin3a	p53 stabilizer	Prevented or inverted pulmonary hypertension in an in vivo model	experimental	Inducer of senescence	[366,367,368]
Dexamethasone	SIRT1 inhibition and p53/p21^WAF/CIP1^ activation	Increased percentage of senescent tenocytes in vitro and in vivo	Anti-edematous, anti-inflammatory	Inducer of senescence	[369]

## Abbreviations

Aβ	amyloid-beta
AD	Alzheimer’s disease
Akt	protein kinase B
ALL	acute lymphocytic leukemia
APP	amyloid beta precursor protein
ATF6α	activating transcription factor 6 isoform α
ATM	ataxia-telangiectasia mutated
BBB	blood-brain barrier
Bcl-2	B-cell lymphoma 2
CDK	cyclin-dependent kinase
CDKI	cyclin-dependent kinase inhibitor protein
cGAS	cyclic GMP-AMP synthase
CIP/KIP	CDK interacting protein/kinase inhibitory protein
CML	chronic myelogenous leukemia
CNS	central nervous system
CSF	cerebrospinal fluid
DDR	DNA damage response
Ecrg4	esophageal cancer-related gene 4
EGF	epidermal growth factor
ER	endoplasmic reticulum
FGF-2	fibroblast growth factor 2
GLB1	galactosidase beta 1
HLA-DR	human leukocyte antigen-DR isotype
γH2AX	phosphorylated H2A histone family member X
IkB	inhibitor of kappa B
IL	Interleukin
MBP	myelin basic protein
MECP	methyl-CpG-binding protein
MMP	matrix metalloproteinase
MRI	magnetic resonance imaging
mTOR	mammalian target of rapamycin
MS	multiple sclerosis
NAWM	normal appearing white matter
NF-κB	nuclear factor kappa-light-chain-enhancer of activated B cells
NSCs	neural stem cells
OIS	oncogene-induced senescence
OPC	oligodendrocyte progenitor cell
PD	Parkinson’s disease
PS1	presenilin-1
p38MAPK	p38 mitogen-activated protein kinases
RB	Retinoblastoma
ROS	reactive oxygen species
RR-MS	relapsing-remitting multiple sclerosis
SA-β-Gal	senescence-associated β-galactosidase
SAHF	senescence-associated heterochromatin formation
SAMD	senescence-associated mitochondrial dysfunction
SASP	senescence-associated secretory phenotype
SIPS	stress-induced premature senescence
SIRT	Sirtuin
SP-MS	secondary progressive multiple sclerosis
SGZ	subgranular zone
STING	stimulator of IFN genes
SVZ	subventricular zone
S1P	sphingosine 1-phosphate receptor
TCDD	2,3,7,8-Tetrachlorodibenzo-p-Dioxin
Terc	human telomerase gene
TGF-β	transforming growth factor beta
TNF-α	tumour necrosis factor alpha
UPR	unfolded protein response
53BP1	p53-binding protein 1
